# Supplementary material for: Nutritional Status Assessed with Objective Data Assessment Correlates with a High-Risk Foot in Patients with Type 2 Diabetes
Source: J Clin Med. 2022 Feb 27;11(5):1314. doi: 10.3390/jcm11051314 (PMC8911330; doi:10.3390/jcm11051314)
Supplement: Supplementary file 1 [file jcm-11-01314-s001.zip › jcm-1585725-supplementary.pdf]

Supplemental Table S1. (**a**). Multivariate-adjusted ORs (95% CI) for high-risk diabetic foot assessed with IWGDF in patients taking statin. (**b**). Multivariate-adjusted ORs (95% CI) for high-risk diabetic foot assessed with IWGDF in patients not taking statin.

(**a**)

	years <75		years ≥75	
	OR (95%CI)	*P*	OR (95%CI)	*P*
Age	1.03 (0.99–1.08)	0.140	1.26 (1.00–1.69)	0.048
Male	2.52 (1.11–6.00)	0.027	3.74 (0.52–45.2)	0.198
Duration of type 2 diabetes	1.04 (0.99–1.08)	0.106	1.07 (0.98–1.18)	0.160
BMI	1.03 (0.94–1.13)	0.508	1.10 (0.80–1.55)	0.558
Hemoglobin A1c	1.15 (0.82–1.65)	0.418	0.81 (0.26–2.44)	0.709
Creatine	1.01 (1.00–1.03)	0.044	1.00 (0.97–1.05)	0.858
Hypertension	1.16 (0.45–3.07)	0.753	1.12 (0.16–8.40)	0.905
Current smoking	1.83 (0.56–5.89)	0.306	2.37 (0.03–30.8)	0.680
CONUT	1.02 (0.82–1.26)	0.866	1.72 (1.03–3.26)	0.034

(**b**)

	years <75		years ≥75	
	OR (95%CI)	*P*	OR (95%CI)	*P*
Age	1.06 (1.02–1.11)	0.006	1.21 (1.00–1.52)	0.046
Male	1.34 (0.59–3.04)	0.480	3.17 (0.56–25.1)	0.196
Duration of type 2 diabetes	1.00 (0.97–1.04)	0.716	1.07 (1.00–1.16)	0.038
BMI	0.95 (0.88–1.03)	0.246	1.08 (0.86–1.39)	0.509

Hemoglobin A1c	1.37 (0.98–1.92)	0.066	0.84 (0.33–2.03)	0.697
Creatine	1.02 (1.01–1.04)	0.001	1.00 (0.97–1.03)	0.966
Hypertension	1.73 (0.77–3.97)	0.181	2.28 (0.43–12.5)	0.329
Current smoking	1.85 (0.51–6.49)	0.339	2.85 (0.10–9.58)	0.552
CONUT	0.90 (0.74–1.10)	0.326	1.64 (1.03–2.87)	0.036

OR, odds ratio; CI, confidence interval; IWGDF, International Working Group on the Diabetic Foot; BMI, body mass index; CONUT, controlling nutritional status.
